# Differential Impact of Emotion on Semantic Processing of Abstract and Concrete Words: ERP and fMRI Evidence

**DOI:** 10.1038/s41598-019-50755-3

**Published:** 2019-10-08

**Authors:** Sophie Pauligk, Sonja A. Kotz, Philipp Kanske

**Affiliations:** 1 Division of Psychological and Social Medicine and Developmental Neuroscience, Faculty of Medicine, Technische Universität Dresden, Dresden, Germany; 2 Research Group Social Stress and Family Health, Max Planck Institute for Human Cognitive and Brain Sciences, Leipzig, Germany; 3 Faculty of Psychology and Neuroscience, Department of Neuropsychology & Psychopharmacology, Maastricht University, Maastricht, The Netherlands; 4 Department of Neuropsychology, Max Planck Institute for Human Cognitive and Brain Sciences, Leipzig, Germany; 5 Clinical Psychology and Behavioral Neuroscience, Faculty of Psychology, Technische Universität Dresden, Dresden, Germany

**Keywords:** Language, Prefrontal cortex, Human behaviour

## Abstract

Emotional valence is known to influence word processing dependent upon concreteness. Whereas some studies point towards stronger effects of emotion on concrete words, others claim amplified emotion effects for abstract words. We investigated the interaction of emotion and concreteness by means of fMRI and EEG in a delayed lexical decision task. Behavioral data revealed a facilitating effect of high positive and negative valence on the correct processing of abstract, but not concrete words. EEG data yielded a particularly low amplitude response of the late positive component (LPC) following concrete neutral words. This presumably indicates enhanced allocation of processing resources to abstract and emotional words at late stages of word comprehension. In fMRI, interactions between concreteness and emotion were observed within the semantic processing network: the left inferior frontal gyrus (IFG) and the left middle temporal gyrus (MTG). Higher positive or negative valence appears to facilitate semantic retrieval and selection of abstract words. Surprisingly, a reversal of this effect occurred for concrete words. This points towards enhanced semantic control for emotional concrete words compared to neutral concrete words. Our findings suggest fine-tuned integration of emotional valence and concreteness. Specifically, at late processing stages, semantic control mechanisms seem to integrate emotional cues depending on the previous progress of semantic retrieval.

## Introduction

Human language encompasses the remarkable ability to communicate about abstract semantics. In the absence of perceptual information, the representation of abstract words must strongly rely on verbal associations. It has been suggested that emotion is differentially involved in the conceptual grounding of abstract and concrete words. The present study will investigate that supposition.

### Concreteness effects

Words can be classified as either concrete or abstract. In general, concrete words are associated with actual *things* and are processed faster and more accurately than abstract words, which are more conceptual. Two mechanisms seem to contribute to the concreteness effect. First, concrete words evoke the retrieval of perceptual in addition to verbal representations^1,2^. Second, abstract words are more contextually diverse and ambiguous^3^, which hinders the retrieval of the semantic context^4–6^. The importance of mental imagery and context availability has been shown behaviourally^7^, with event-related potentials (ERP)^8,9^, and with functional magnetic resonance imaging (fMRI)^10^. In comparison to abstract words, concrete words typically elicit increased activation in left parietal and occipital brain areas involved in mental imagery^11,12^. In contrast, abstract words activate two central nodes of the language processing network: the left middle temporal gyrus (MTG) and left inferior frontal gyrus (IFG). The MTG is a key region for lexico-semantic representation and retrieval^13,14^ whereas the IFG is central to context-sensitive control of lexico-semantic processing^14–17^. While the fMRI findings clearly highlight the importance of mental imagery and context availability, the interpretation of ERP concreteness effects is still under debate.

Early ERP studies have shown that context availability influences the N400 concreteness response, a negative deflection observed at 300–550 ms post-stimulus with a more anterior distribution than the more common semantic N400^8^. Mental imagery may be linked to the late positive component (LPC, also referred to as the ‘N700’), a positive-going ERP observed at 550–800 ms^9,18^. Compared to abstract words, concrete words typically display more negative-going N400 and LPC responses. Recent findings contest interpretations of N400 and LPC concreteness effects that are based on context availability and word imageability. First, significant N400 and LPC effects were observed despite controlling for the two variables^19^. Second, semantically rich concrete words (with a higher number of semantic associations) elicited an N400 response that is similar to abstract words. This contradicts an association between N400 and activation of semantic context^20,21^. Third, source localization pinpointed the origin of N400 and LPC concreteness effects to the left IFG^22^. Building on this finding, Adorni and Proverbio^22^ proposed that N400 and LPC concreteness effects are both linked to the strategic control of semantic retrieval, realized in the IFG. This notion may provide a coherent explanation for the ERP results reviewed above. More positive-going ERP responses (lower N400 amplitude, larger LPC amplitude) could be caused by an increase in semantic control. This may stem from a lack (in the case of abstracts words) or surplus of lexico-semantic information (for semantically rich concrete words, as in Amsel & Cree^20^ and Kounios *et al*.^21^). Also, an increased need for semantic control may underlie other factors than imageability and context availability (e.g. Barber *et al*.^19^). Sentence comprehension studies support this idea, as highly complex or ambiguous contexts lead to larger late anterior LPC responses^8,23,24^.

### Role of emotion in concreteness?

How valid current explanations of the concreteness effect are is further challenged by abstractness effects. Studies that controlled for a word’s imageability and context availability^19,25,26^ revealed better performance for abstract than concrete words. In these studies, abstractness effects may be driven by higher emotional valence of abstract words^26^. Highly emotional words (i.e., positive or negative) are processed faster and more accurately than neutral words^27^. However, emotional valence also interacts with concreteness if the two factors are manipulated orthogonally^18,28^. These findings reach beyond a statistical predominance of emotional features in abstract words. Rather, they point towards a functional difference in the way emotional valence affects the processing of concrete and abstract words.

### Emotion effects

ERP and fMRI studies demonstrate that emotion affects early as well as late word processing stages (for comprehensive reviews, see Citron^29^ and Kissler, Assadollahi, & Herbert^30^). In addition to very early emotion effects (e.g. on the P2^31^), there are two emotion-sensitive ERP components that temporally overlap with ERP concreteness effects. The N400 is thought to reflect facilitated lexico-semantic retrieval and contextual integration of emotional words^32–34^. The LPC, which usually shows larger amplitudes for emotional than neutral words^18,35,36^, mirrors increased allocation of capacity-limited resources for detailed stimulus evaluation and response preparation, based on task demands^29,37^. fMRI studies report emotion-related activation increases in the amygdala^38,39^, parahippocampal gyrus^40,41^, ventral anterior cingulate cortex (ventral ACC^42,43^), orbitofrontal cortex^44,45^, and dorsolateral and medial prefrontal cortices^46,47^. The functions of these regions range from automatized integration of perceptual information to higher-order, cognition-based evaluation processes. According to the grounded cognition framework^48^, word processing may involve the joint activation of language and emotion areas. This is explained by the inextricable links between a concept’s linguistic representation and associated emotional aspects, physiological responses and motor correlates. For instance, processing the word “bravery” may include the partial neural re-enactment of perceptual and emotional aspects of the state it refers to. Investigating interactions between emotion and other lexico-semantic variables (such as concreteness) will allow the further specification of spatial-temporal and conceptual properties of emotion’s role in semantic processing.

### Interaction of concreteness and emotion

Orthogonal manipulations of emotion and concreteness have, to date, yielded mixed results. Two ERP studies using lexical decision tasks (LDTs) support an amplification of the concreteness effect by emotion. A hemifield go-/no-go LDT by Kanske and Kotz^18^ yielded a significant effect of emotion on the LPC for concrete words only. Based on previous findings^9^, this effect was attributed to the differential engagement of mental imagery by emotional concrete words. Given current knowledge, the effect may also be explained by a high level of semantic richness and the enhanced need for semantic control elicited by emotional concrete words^20–22^. In an LDT with verbs, Palazova *et al*.^28^ found an effect of emotion at 250–300 ms that occurred for concrete words only. The authors attributed this effect to a slower access of semantic information for abstract words. The absence of an interaction in the LPC time window might be explained by the experimental design – non-delayed LDTs can prevent findings in the LPC time window due to simultaneously occurring behavioral responses^18^. Compared to nouns, the use of verbs more strongly triggers the automatic access of syntactic information^49^, which might also affect observations in the LPC time window^50^.

A different theoretical approach is inspired by the grounded cognition theory^51^. It states that internal representation and processing relies on the same neural mechanisms as action and perception. As abstract concepts cannot likely be embodied via sensory or motor information, internal affective experience constitutes a probable alternative. Accordingly, Vigliocco, Meteyards, Andrews, and Kousta^52^ propose a preponderance of emotional features in the representation of abstract words. However, their suggestion that the rostral ACC may be involved in emotion-sensitive processing of abstract words^26^ is called into question by a fully crossed factorial investigation which did not show an interaction between emotion and concreteness in this region^53^. Yet, to our knowledge, a stronger involvement of emotional valence in the processing of abstract words is supported by two studies, both of which used an orthogonal design. In an ERP concreteness judgment task, Kaltwasser, Ries, Sommer, Knight, and Willems^54^ reported stronger LPC emotion effects for abstract words. Secondly, an eye tracking study by Sheikh and Titone^55^ showed that higher emotional valence strongly facilitated the reading of abstract rather than concrete words.

### The present study

The present study aims to systematically manipulate concreteness and emotion in an LDT to pursue the following aims. First, by avoiding factors that have limited previous ERP studies, we aim to broaden our understanding of the electrophysiological correlates related to emotion and concreteness. Second, the study aims at localizing the interaction of emotion and concreteness by means of a factorial, whole brain fMRI analysis – an experimental set-up that, to our knowledge, has not yet been tested. More specifically, the present study replicates Kanske and Kotz^18^ in a non-hemifield design. This is important with regard to the generalizability of ERP effects. A delayed response design is employed to capture lexico-semantic processing without response interference in the LPC time window (as in Palazova *et al*.^28^) or response inhibition inherent in a go/no-go task (as in Kanske & Kotz^18^). By utilizing implicit processing of concreteness, it is guaranteed that the observed effects do not reflect conscious decision making processes or design characteristics of a concreteness judgment task, as in Kaltwasser *et al*.^54^. As there is substantial evidence that LDTs evoke semantic processing^56^, the terms ‘lexico-semantic’ and ‘semantic’ processing will be used interchangeably to describe the processing of written word stimuli in this type of task. With regard to the interaction of emotion and concreteness, there are two competing hypotheses that may apply to behavioral as well as ERP and fMRI signal variance.

1. Differential activation of mental imagery by concrete and abstract words will lead to an amplification of emotion effects for concrete words.
2. Due to stronger emotional grounding^52^ of abstract versus concrete words, the emotion effect will be amplified in abstract words.

fMRI effects could, according to hypothesis (1), reflect enhanced neural activity in brain areas associated with mental imagery, for instance left parietal and occipital areas^11,12^, or the left basal temporal cortex^57^. According to an embodied cognition view (hypothesis (2)), an interaction of emotion and concreteness could emerge within areas primarily known as emotion-related, or from the IFG. The later aids in the resolving of semantic ambiguity by retrieving an appropriate semantic context^14^, while also being sensitive to emotional valence^10,58^. According to hypothesis (1), a significant ERP interaction would be expected in the LPC time-window. In the context of hypothesis (2), an interaction in the N400 or LPC time window is plausible.

## Experimental Procedures

### Rating and stimuli

Word stimuli were identical to those used by Kanske and Kotz^18^ and based on a previous rating study^59,60^. The set of words included 240 nouns, half rated as abstract, the other half as concrete. Emotion was operationalized by emotional valence. Neutral words (50% of the stimuli) were contrasted with emotional words (negative and positive, 25% each). Arousal ratings were comparable for positive and negative words, but differed between emotional and neutral words. The stimuli did not include words that describe emotions (e.g. “fear”). To allow for a fully crossed factorial design, the subsets of concrete and abstract words each contained an equal number emotional and neutral words. An analysis of variance (ANOVA) confirmed significant effects for emotional valence, arousal, and concreteness, but not for word length, frequency of usage, and familiarity (for statistical details, see Supplementary Information (SI), Paragraph 1). We chose not to control for age of word acquisition (AoA), although it is naturally higher in abstract compared to concrete words^25^. AoA is highly correlated with word frequency, length, and imageability^61^ and some authors thus question independent effects of AoA on word reading^62^. Experiments that controlled for word length, frequency, familiarity, imageability, and AoA simultaneously yielded comparatively artificial sets of concrete and abstract words, which also differed in valence^25^. We therefore chose to carefully control for word frequency and length only, opting for a more natural stimulus set at the expense of psycholinguistic purity^63^. Pseudowords (240) were created by substitution of one letter in each word of the word set. The phonological rules of German were followed in this process. For detailed information about word and pseudoword properties, see SI, paragraph 1. The same set of words was used in the electroencephalography (EEG) and the fMRI experiment.

### EEG-Experiment

#### Participants

Thirty students of the University of Leipzig participated in the study and were paid for their participation (8€/h). The sample excluded participants of the rating study and a previous study with the same stimulus material^18^. Three participants of the original sample were excluded from further data analysis because rejection of artifacts had reduced the number of valid trials in at least one condition by more than one third. The final sample included 27 participants (14 women); mean age was 24.4 years (standard deviation (SD) = 2.2). All participants were right-handed according to the Edinburgh Handedness Inventory^64^ and reported normal or corrected-to-normal vision. Participants for the EEG and the fMRI experiment agreed to participate in the study after providing written informed consent. The protocol for both experiments was approved by the Ethics Committee of the University of Leipzig and carried out in line with the Declaration of Helsinki.

#### Task and procedure

The participants completed a delayed lexical decision task (LDT), indicating whether a visually presented stimulus was a word or a pseudoword by left and right button presses. Assignment of responses to keys was counterbalanced across participants. Each trial started with the presentation of a jittered fixation cross (duration: 0, 500, 1000, or 1500 ms). Afterwards, the target stimulus (lexical word or pseudoword) was presented centrally for 200 ms. It was followed by another fixation cross, which turned red after 800 ms to signal the onset of the response window. The response window ended upon the participants’ response or after a maximum of 2500 ms. Subsequently, the fixation cross turned black again for 2000 ms. The overall duration of one trial varied between 3000 and 7000 ms. After arrival and application of the EEG-cap, participants were instructed and completed four practice blocks. The first practice block consisted of 10 while the last three had 20 trials each. If less than 60% of all practice trials had been answered correctly, practice was repeated until answers were correct in at least 60% of the trials. The main experiment consisted of four blocks of 120 trials each. Hence, all target stimuli were presented once.

Positive and negative words were presented in separate blocks, each with an equal number of neutral counterparts. The order of the positive and negative blocks was counterbalanced across the sample. This allowed to separately investigate effects of positive and negative valence (as in Kanske & Kotz^18^) while avoiding stimulus proportion effects in the ERPs^65,66^. Notably, this kind of block design may bias reaction time (RT) comparisons between positive and negative stimuli^67,68^, a trade-off we accepted as the behavioral data were of reduced informative value (due to the delayed response design) and not the focus of our analyses. The overall duration of the EEG-recording was about 40 min. The experiment was programmed and run in ERTS (Experimental Run Time System, ERTSlab, 1999). All stimuli were presented on a computer screen with a viewing distance of approximately 100 cm. With the exception of the red fixation cross that signaled the response option, all stimuli were presented in black on a light gray background.

#### EEG Recording

64 Ag-AgCl electrodes were applied according to the international 10–20 system of EEG recording^69,70^. The ground electrode was placed at the sternum. The left mastoid served as a reference, whereas the right mastoid was recorded actively. After recording, data were re-referenced offline to the average of both mastoids. Impedances were below 5 kΩ for all recordings. Lateral electrodes recorded horizontal electrooculograms on both eyes; vertical eye movements were measured with two electrodes above and below the right eye. The recording was done with Brain vision professional recorder software (Brain Products GmbH, Munich, Germany) and the corresponding amplifier Brainamps. Sampling rate was 500 Hz. Recorded data were filtered online with a bandpass between DC and 250 Hz. A lowpass filter (7 Hz) was applied to correct for graphical display only.

#### Data analysis

Performance data, grand averages of EEG data and mean extracted beta values from the fMRI data set (described below) were analyzed with the SPSS software package (Statistical Package for the Social Sciences, Version 22.0, IBM Corp., Armonk, NY). Incorrect trials were excluded from RT analysis, EEG, and fMRI analyses. Univariate repeated measures ANOVAs were conducted with error rates, RTs, ERP data, and fMRI data. Behavioral data of the EEG and the fMRI sample (which followed an equivalent experimental setup, see below) were evaluated in a single analysis. The mode of data acquisition (EEG versus fMRI) was included in a mixed design ANOVA as a between subjects factor. The factor did not yield a significant main effect nor any significant interactions. A separate analysis of positive and negative words did not yield any significant main effects of emotion (positive, negative) or interactions between emotion and concreteness in the fMRI analysis and the three ERP component analyses of interest (P2, N400, LPC). Thus, to enhance statistical power, we pooled positive and negative stimuli, using a single emotional condition for all analyses reported here. Emotion (emotional, neutral) and concreteness (concrete, abstract) served as factors in the ANOVAs of the behavioral, EEG, and fMRI data. For the sake of brevity, EEG and fMRI main effects are only reported when significant. Interactions are only described if they yielded significant follow-up analyses. Follow-up analyses for main effects and interactions were carried out as simple effect analyses and pairwise comparisons. For all ANOVAs, Greenhouse-Geisser corrected F-values are provided^71^. η^2^ is reported as a measure of effect size^72^.

#### EEG Analysis

Preprocessing of EEG data was performed with the EEP software package (Evaluation Package EEP 3.2, Copyright © Max Planck Institute for Human Cognitive and Brain Sciences, Leipzig, Germany). Quantification of ERPs was achieved by measuring mean amplitudes during an epoch of 1000 ms after stimulus onset, relative to a 200 ms pre-stimulus baseline. Trials that exceeded 30 μV for the two eye channels or 40 μV for CZ and PZ within a sliding window of 200 ms were automatically rejected from the analysis. Furthermore, all trials were manually checked for artifacts. The percentage of remaining trials was calculated for each participant separately. Participants were excluded from subsequent analyses if rejection of artifacts had reduced the trial number in at least one ANOVA condition by more than one third. This procedure ensured that each cell of the ANOVA included at least 40 trials per participant. Average ERPs were computed for each condition and participant, and for each condition across participants (grand average).

In order to analyze the topographic distribution of the effects of interest (cf. Kanske & Kotz^18^; Palazova *et al*.^28^), we chose a grouping of the channels in four regions of interest with eleven electrodes each (left anterior: FP1, AF3, AF7, F3, F5, F7, F9, FC3, FC5, FT7, and FT9; right anterior: FP2, AF4, AF8, F4, F6, F8, F10, FC4, FC6, FT8, and FT10; left posterior: CP3, CP5, TP7, TP9, P3, P5, P7, P9, PO3, PO7, and O1; right posterior: CP4, CP6, TP8, TP10, P4, P6, P8, P10, PO4, PO8, and O2). In addition to the factors emotion and concreteness, the ANOVAs of EEG data included the within-subject factors region (anterior and posterior) and hemisphere (left and right), allowing for comparisons between the four regions of interest. As effects of lateralization were not significant, main effects of hemisphere and region as well as their interaction are reported in the SI only (paragraph 2).

### fMRI Experiment

#### Participants

Twenty-one students of University of Leipzig (11 female) volunteered for this experiment and were paid for their participation (8€/h). The sample excluded participants of the rating study, the hemifield study^18^, and the EEG study. Mean age of the sample was 23.3 years (SD = 1.9). All participants reported normal or corrected-to-normal vision and were strongly right-handed according to The Edinburgh Handedness Inventory^64^.

#### Task and procedure

Task and procedure closely resembled those of the EEG study. The setup was identical except for the overall trial duration. To ensure sufficient distance between stimuli, the overall length of trials in the fMRI study was fixed to 6 seconds by adapting the duration of the last fixation cross, according to the varying length of the rest of the trial. In the EEG trials, the duration of the last fixation cross was fixed at 2000 ms, leading to variations in the overall trial length. Participants stayed in the MRI scanner for about 45 minutes. They were instructed to relax and move as little as possible. The experiment was programmed and run in ERTS (Experimental Run Time System, ERTSlab, 1999). Participants viewed the stimuli on a back-projection screen with the aid of a mirror-based system; overall viewing distance was about 100 cm.

#### fMRI Data acquisition

fMRI data collection was performed on a 3.0-Tesla scanner (Bruker 30/100 Medspec system, Bruker Medizintechnik GmbH, Ettlingen, Germany) at the Max Planck Institute for Human Cognitive and Brain Sciences in Leipzig (Germany). A standard birdcage head coil was used. A high-resolution whole-head 3D Modified Driven Equilibrium Fourier Transform brain scan was acquired (128 sagittal slices, 1.5-mm thickness, FOV 25:0 × 25:0 × 19:2 cm, data matrix of 256 × 256 voxels) in order to exclude participants with neurological anomalies. Scout spin echo sagittal scans were collected to define the anterior and posterior commissures on a midline sagittal section. Afterwards, structural and functional (echo-planar) images were obtained from 24 axial slices parallel to the plane intersecting the anterior and posterior commissures (AC–PC plane). The whole range of slices covered almost the entire brain. For functional imaging, a gradient echo planar imaging (EPI) sequence was used (repetition time: 2.0 sec, echo time: 30 msec; 3 images per trial, flip angle: 90°, acquisition bandwidth: 100 kHz). The matrix acquired contained 64 × 64 voxels with an FOV of 19.2 cm, resulting in an in-plane resolution of 3 × 3 mm. The slice thickness was 4 mm with an interslice gap of 1 mm.

#### fMRI Data analysis

Imaging data were analyzed using SPM 8 (Statistical Parametric Mapping software, Wellcome Trust Centre for Neuroimaging, http://www.fil.ion.ucl.ac.uk/). Functional data were re-aligned, slice-time corrected, and normalized to a standard EPI template. A spatial Gaussian filter with 8-mm full-width at half-maximum was applied for smoothing. A temporal high-pass filter with a cut-off frequency of 1/100 Hz was used to remove low-frequency variations in the blood oxygen level dependent (BOLD) signal. In order to improve motion correction, first level contrasts were specified with the rWLS toolbox^73^. Individual statistical parametric maps were calculated to elucidate: (1) emotion effects (emotional vs. neutral words), (2) concreteness effects (concrete vs. abstract words).

The individual participants’ data were then analyzed using a General Linear Model for BOLD signal changes due to the experimental conditions. Concreteness (concrete, abstract) and emotion (emotional, neutral) served as factors in a univariate repeated measures ANOVA. In a separate SPM analysis, a direct contrast of positive and negative stimuli yielded no significant differences, thus supporting the pooling of the two emotion conditions. Correction for multiple comparisons was accomplished by a combination of individual voxel probability thresholding and minimum cluster size thresholding in 3DClustSim (http://afni.nimh.nih.gov/pub/dist/doc/program_help/3dClustSim.html – updated version, released August 2016). Based on Monte Carlo simulations, a minimum cluster size of k = 53 was calculated to ensure a given alpha level of false positive findings (α = 0.05, two-sided) for a given voxelwise p-value level (p = 0.001). Follow-up analyses for main effects and interactions were carried out as simple effect analyses and pairwise comparisons of mean percent BOLD signal change. Behavioral and EEG data are publicly available via the Open Science Framework (https://osf.io/hcujt/). Unthresholded 2^nd^ level fMRI contrasts can be retrieved via Neurovault (https://identifiers.org/neurovault.collection:5922).

## Results

### Performance data

EEG and fMRI performance data were evaluated in a single analysis, thus containing all participants from the two final samples (n = 48). When included into a mixed design ANOVA, the mode of data acquisition (EEG, fMRI) did not yield a significant main effect or significant interactions. The majority of trials were answered correctly (95.3%, SD = 4.0%). Mean RT across all trials was 388.5 ms (SD = 189.3). Compared with abstract words, concrete words yielded shorter response latencies (mean = 391.5 (SD = 107.2) versus mean = 383.2 (SD = 105.1), F(1,47) = 6.49, P < 0.05, η2 = 0.12) as well as lower error rates (mean = 5.7 (SD = 4.6) versus mean = 3.5 (SD = 4.2), F(1,47) = 31.79, P < 0.001, η2 = 0.40). There was no significant effect of emotion with regard to RTs, but error rates were significantly lower for emotional compared to neutral words (mean = 4.0 (SD = 4.3) versus mean = 5.2 (SD = 4.5), F(1,47) = 11.17, P < 0.01, η2 = 0.19). Error rate analysis also showed a significant interaction between emotion and concreteness (F(1,47) = 6.77, P < 0.05, η2 = 0.13). The difference between emotional and neutral words was only significant for abstract (F(1,47) = 15.55, P < 0.001, η2 = 0.25), but not for concrete words, with particularly high error rates in the neutral abstract condition. Means and SDs of error rates and RTs can be seen from Table 1.

**Table 1:** Performance data.

	Mean RTs in ms (SD)		Mean Error Rates in % (SD)	
	Abstract	Concrete	Abstract	Concrete
Neutral	396 (111)	384 (107)	6.7 (5.0)	3.7 (4.6)
Emotional	387 (106)	382 (107)	4.7 (4.8)	3.3 (4.4)
Positive	387 (112)	382 (113)	3.5 (5.1)	2.6 (4.1)
Negative	387 (109)	382 (105)	5.9 (5.4)	4.0 (5.7)

Means and standard deviations (in parentheses) of reaction times and error rates.

### EEG Results

**Figure 1:**
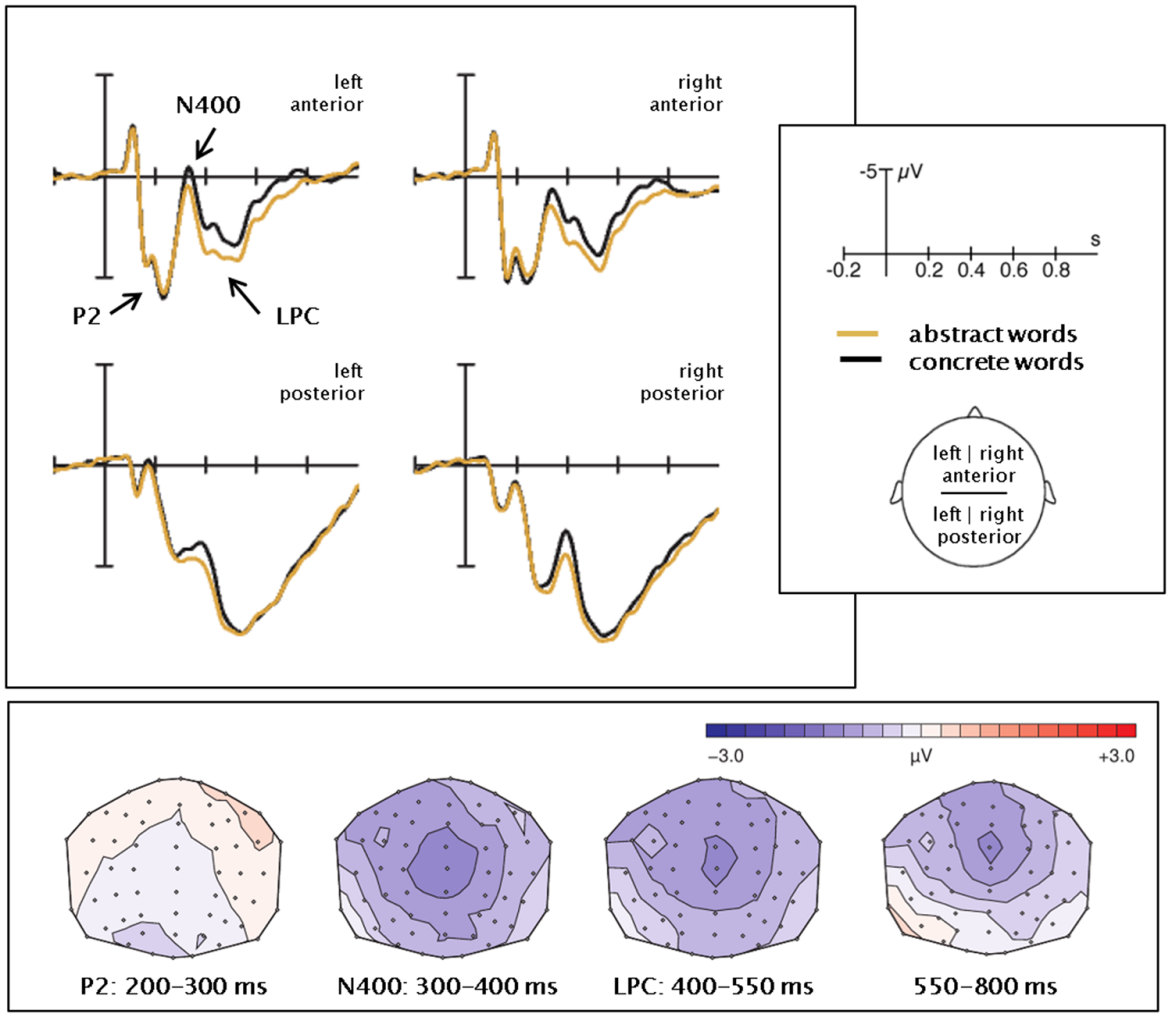
EEG concreteness effects. Average ERPs in the four ROIs. The difference maps were obtained by subtraction of the abstract from the concrete ERPs.

**Figure 2:**
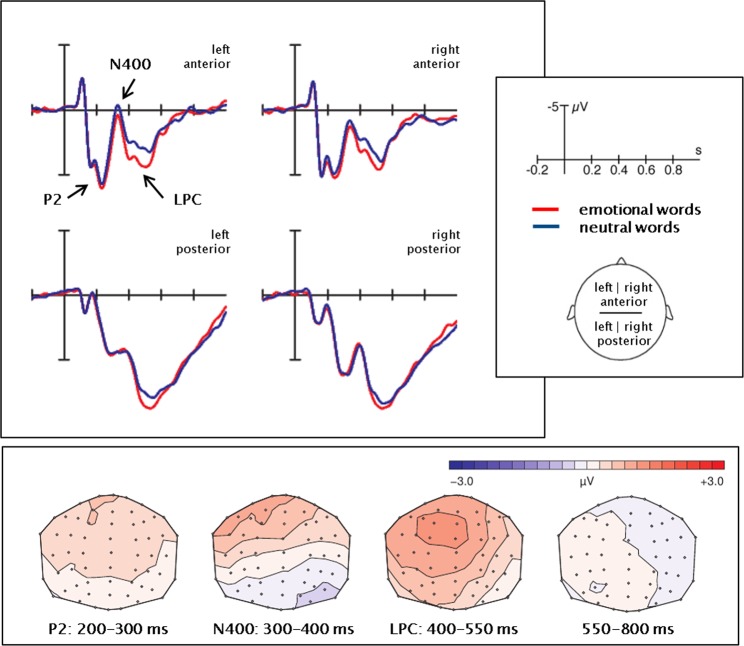
EEG emotion effects. Average ERPs in the four ROIs. The difference maps were obtained by subtraction of the neutral from the emotional ERPs.

**Figure 3:**
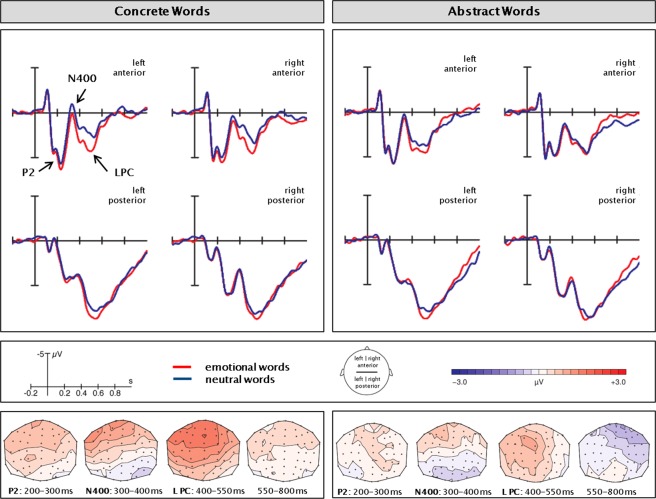
Interaction concreteness * emotion in the EEG data. Average ERPs in the four ROIs. The difference maps were obtained by subtraction of the neutral from the emotional ERP measurements.

ERPs elicited by concrete and abstract words are displayed in Fig. 1 while ERPs for emotional and neutral words can be seen in Fig. 2. Figure 3 illustrates the interaction between concreteness and emotion by displaying the emotion effect separately for concrete and abstract words. A time-line analysis of 50 ms segments allowed for an overview of ERP effects – see Table 2. Descriptively, an N1 (peak around 100 ms) and a P1 (peak around 150 ms) were followed by another positive peak around 230–260 ms, an N400 (peak at 320–370 ms), and an LPC (420–550 ms). Main effects of concreteness occurred from 300–600 and 650–800 ms after stimulus onset (Fs(1,26) ≥ 4.94, Ps ≤ 0.04, all η^2^ ≥ 0.16). Emotion significantly influenced ERP amplitudes at 250–350, 400–550, and 700–750 ms after stimulus onset (Fs(1,26) ≥ 4.97, P ≤ 0.03, all η^2^ ≥ 0.16). An interaction between concreteness and emotion was observed in the interval 450–500 ms after stimulus onset (F(1,26) = 5.21, P ≤ 0.03, η^2^ = 0.17). Based on visual inspection of ERP developments and the time-line analysis, we decided to further explore the effect of three ERP components. Repeated measures analyses were conducted for the latency intervals of 200–300 ms (P2 component), 300–400 ms (N400 component), and 400–550 ms (LPC). Due to the central peak of the concreteness effects, we ran an identical set of analyses including the central electrodes (see SI, paragraph 3). Observing an identical patterns of results, we chose to report the set-up with larger spatial coverage and better comparability to our previous work.

**Table 2:** EEG time-line analysis.

time after stimulus onset (ms)	200–250	250–300	300–350	350–400	400–450	450–500	500–550	550–600	600–650	650–700	700–750	750–800
Concreteness			**13.45**	**36.48**	**37.21**	**16.71**	**6.73**	**5.01**	3.15	**4.94**	**9.26**	**5.91**
			**(0.001)**	**(0.000)**	**(0.000)**	**(0.000)**	**(0.02)**	**(0.03)**	(0.09)	**(0.04)**	**(0.01)**	**(0.02)**
			0.34	0.58	0.59	0.39	0.21	0.16		0.16	0.26	0.19
Emotion	3.45	**7.5**	**5.02**		**15.86**	**25.05**	**14.96**			3.16	**4.97**	
	(0.07)	**(0.01)**	**(0.03)**		**(0.000)**	**(0.000)**	**(0.000)**			(0.09)	**(0.03)**	
		0.22	0.16		0.38	0.49	0.37				0.16	
Interaction Con*Emo					3.9	**5.21**		3.81				4.08
					(0.06)	**(0.03)**		(0.06)				(0.05)
						0.17						

Statistical analysis of ERPs (F-Value, p-value in parenthesis, η2) for 12 consecutive time windows of 50 ms duration.

#### P2

Analysis of the mean amplitude yielded a main effect of emotion (F(1,26) = 6.68, P < 0.05, η^2^ = 0.20) with larger amplitudes for emotional than neutral words.

#### N400

Analyses yielded a main effect of concreteness (F(1,26) = 27.69, P < 0.001, η^2^ = 0.52), as concrete words led to a larger N400 than abstract words. There was a significant interaction of emotion and region (F(1,26) = 19.31, P < 0.001, η^2^ = 0.43). The effect of emotion was significant at anterior electrode-sites (F(1,26) = 14.59, P < 0.001, η^2^ = 0.36), with larger N400 amplitudes elicited by neutral than by emotional words. There was no significant effect of emotion at posterior electrode sites (F(1,26) = 1.26, P = 0.27, η^2^ = 0.05).

#### LPC

The LPC was larger for abstract than for concrete words (F(1,26) = 22.03, P < 0.001, η^2^ = 0.46). Emotional words yielded a higher LPC amplitude than neutral words (F(1,26) = 24.75, P < 0.001, η^2^ = 0.49). There was a marginally significant interaction between emotion and concreteness (F(1,26) = 4.1, P = 0.05, η^2^ = 0.14), indicating that the effect of emotion may be larger for concrete than for abstract words. Also, emotion interacted with region and with hemisphere (F(1,26) = 7.19, P < 0.05, η^2^ = 0.22 and F(1,26) = 8.43, P < 0.01, η^2^ = 0.25, respectively). The effect of emotion was larger over anterior compared to posterior electrodes (F(1,26) = 30.21, P < 0.001, η^2^ = 0.54 versus F(1,26) = 9.6, P < 0.01, η^2^ = 0.27) and at the left compared to the right hemisphere (F(1,26) = 29.85, P < 0.001, η^2^ = 0.53 versus F(1,26) = 13.33, P < 0.01, η^2^ = 0.34). Following up on the significant influence of region as main effect (see SI, paragraph 2) and in interaction with emotion, the ANOVA was repeated for anterior and posterior electrodes separately. Results showed unaltered main effects and interactions in both regions, and additionally, a significant interaction between emotion and concreteness in the anterior region (F(1,26) = 10.80, P < 0.01,η^2^ = 0.29). At anterior electrode sites, the effect of emotion was larger for concrete (F(1,26) = 25.24, P < 0.001, η^2^ = 0.58) than for abstract words (F(1,26) = 5.09, P < 0.05, η^2^ = 0.16), yielding especially low LPC amplitudes for neutral concrete words.

### fMRI Results

**Figure 4:**
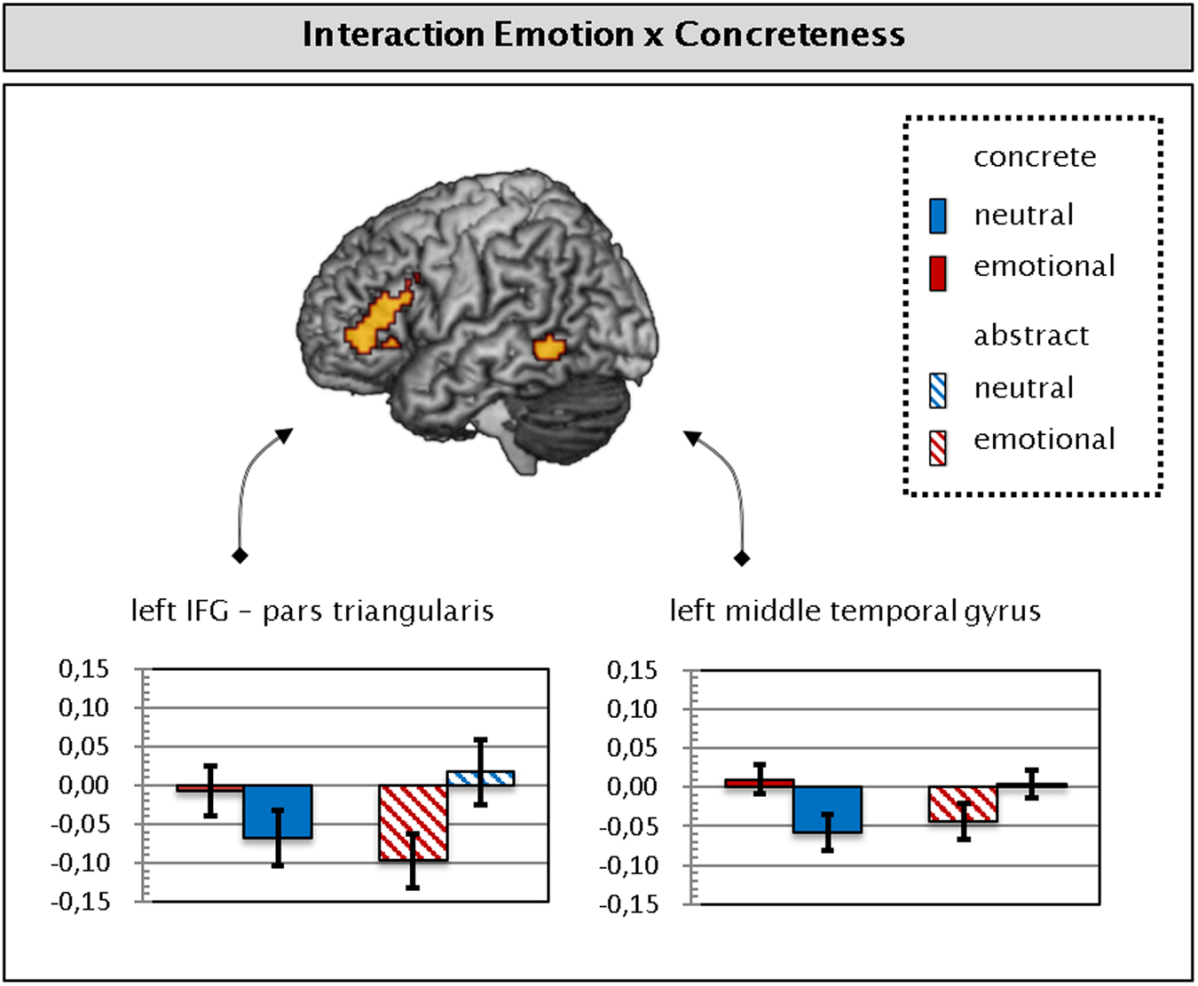
Interaction concreteness * emotion in the fMRI data, mean percent BOLD signal change. Regions surpassing the maximum activation proportion expected under the null hypothesis superimposed on a standard single subject brain. Error bars represent the standard error of the mean.

Topographical and statistical details of significant fMRI peak activations are given in Table 3. Supplementary Fig. 1 depicts the fMRI main effects of concreteness and emotion. In comparison to concrete words, abstract words elicited increased activity in the orbital part of the left IFG. A part of the right middle frontal gyrus was activated in response to neutral as compared to emotional words. The contrasts concrete > abstract and emotional > neutral did not yield any suprathreshold clusters. Figure 4 illustrates the interaction between concreteness and emotion. Analyses revealed a significant interaction in the pars triangularis of the left IFG and a part of the left middle MTG. In both regions, the BOLD signal emotion effect was inversed when comparing concrete with abstract words. The BOLD signal patterns indicated enhanced neural activity in response to emotional concrete and neutral abstract words as compared to neutral concrete and emotional abstract words. Pairwise post-hoc comparisons revealed that the effects of emotion were significant for concrete as well as abstract words in MTG as well as IFG (Fs(1,20) ≥5.68, Ps ≤0.027, all η^2^ ≥ 0.22).

**Table 3:** fMRI peak activations.

Contrast	Brain region	H	x	y	z	K	zmax	BA
*Abstract* > *Concrete*	Orbital Part of IFG	L	−54	20	−5	**76**	3.9	38/45/47
		L	−51	26	−11		3.86	38
*Concrete* > *Abstract*	—							
*Emotional* > *Neutral*	—							
*Neutral* > *Emotional*	Middle Frontal Gyrus	R	39	50	16	**124**	4.22	46
Emotion*Concreteness	Middle Temporal Gyrus	L	−57	−46	−8	**62**	4.08	20/21/37
	Pars Triangularis of IFG	L	−51	41	1	**135**	3.89	45
		L	−54	29	16		3.72	45
		L	−51	14	22		3.49	48

H = hemisphere; x,y,z = MNI coordinates of peak voxel; k = cluster size (number of voxels); zmax = peak z value, BA = Brodmann area; IFG = Inferior Frontal Gyrus.

## Discussion

The present study investigated the interplay of word concreteness and emotional valence in a lexical decision task. We hypothesized to observe either a stronger emotion effect on concrete words (due to the stronger involvement of mental imagery) or a stronger emotion effect on abstract words (due to the stronger emotional grounding). Error rates and MRI findings in the left IFG and left MTG support amplified emotional grounding of abstract words. However, the MRI contrasts of concrete words and the interaction in the LPC component of the EEG data cannot clearly be interpreted in favor of either hypothesis. Instead, they point towards the importance of semantic control mechanisms and the availability of semantic retrieval cues. These interpretations will be discussed in detail below.

### Concreteness effects

Concreteness effects in performance data, the N400 and the LPC replicate previous results^9,18^. The relation of N400/LPC amplitudes to mental imagery and lexico-semantic retrieval remains subject to discussion. From our fMRI results and the scalp distribution of the ERP results, it seems possible that the N400 and LPC concreteness effects both originate in the IFG, as described by Adorni and Proverbio^22^. This would crucially challenge current interpretations of concreteness effects in the N400 (e.g. Holcomb *et al*.^8^) and LPC (e.g. Kanske & Kotz^18^). Holcomb *et al*. attribute the enlarged N400 response of concrete words to the increased need for semantic integration following a heightened co-activation of semantic neighbors. This contradicts a hypothetical origin of the N400 concreteness effect in the IFG, as IFG activation patterns are known to reflect a heightened need for semantic integration in abstract, but not concrete words. Further, the current literature on IFG function does not mention its involvement in mental imagery, which is fundamental to the LPC account proposed by Kanske and Kotz^18^. In the current study, all ERP components displayed a comparatively early time course, with the N400 appearing at 300–400 ms and the LPC appearing at 400–550 ms after stimulus onset. This may be due to low task demands, the central presentation of the stimuli, and the sole recruitment of young, highly proficient students. Additionally, a significant effect of concreteness was found 650–800 ms after stimulus onset, possibly constituting a prolonged LPC concreteness effect^8,28^. Occurring directly prior to the response option onset at 800 ms, it may also represent a response-locked, lateralized readiness potential^74^, which varies with concreteness due to the slower processing of abstract words. Enhanced activation of the left IFG falls in line with abstract words requiring elaborated memory retrieval of semantic information^15,75,76^ and top-down control of semantic selection^14,77^. The peak activation was observed in the posterior part of the IFG, a region that seems to be particularly involved in top-down controlled selection between semantic information and the handling of semantic complexity^17^. In summary, fMRI results confirm the importance of top-down semantic control in the processing of abstract words.

### Emotion effects

The often seen behavioral facilitation effect of emotional words was only significant in the error rates. The absence of an emotion effect in the RTs most likely results from a buffering of speed advantages caused by the delayed response design. The N400 and the LPC data indicate in-depth processing of emotional valence despite the absence of an RT effect. Early (P2) as well as late (N400 and LPC) ERP emotion effects replicate previous findings. They suggest that emotional words are processed with higher early selective^31,34^ and sustained attention^37^ as well as enhanced resources for stimulus evaluation at later semantic processing stages^78^. The replication of the N400 emotion effect^79,80^ emphasizes the complexity of lexico-semantic processing at this stage. It affirms van Berkum’s notion^81^ that a multitude of semantic factors may act as lexico-semantic retrieval cues, for instance the interpretive context and relevance signals such as emotional connotation. Additionally, a significant main effect of emotion was found at 700–750 ms. It might reflect a response-locked, lateralized readiness potentials that varies with emotional valence^82^. Alternatively, it could be part of a long-lasting slow wave positivity linked to memory encoding and top-down influences on emotion evaluation^78^. The fMRI data suggest that the right middle frontal gyrus, a part of the dlPFC, was selectively involved in the processing of neutral words. This area regulates inhibitory processes^83^ and sustained attention^84^ and is sensitive to emotional valence in cognitive tasks^85,86^ with mixed polarity regarding emotional valence. That is, enhanced activation is reported in response to neutral^45^ as well as emotional words^41,46^. In the current study, a comparatively long and monotonous procedure may have increased the need for control of sustained attention, which could, due to their lower intrinsic salience, have been particularly high for neutral words.

### Interaction effects

Error rate data yielded a facilitation effect of high emotional valence on abstract, but not concrete words. This is in accordance with emotional valence playing a more pronounced role in the grounding of abstract words. Accordingly, the processing of neutral abstract words may be especially prone to errors because it can neither be based on re-enactment of perceptual nor of emotional states.

In the current data, as well as in similar studies^18^, a lack of coherence between behavioral performance and LPC amplitude differences constitutes an obstacle in interpreting interactions between emotion and concreteness. Improved performance for concrete compared to abstract words is accompanied by smaller LPC responses, whereas improved performance of emotional compared to neutral words is associated with larger LPC responses. Consequently, a conceptual explanation of the behavioral effects cannot straightforwardly relate to LPC amplitude differences. Therefore, our interpretation builds on a more resource-oriented LPC account. Regarding emotion effects, larger LPC amplitudes are associated with the increased allocation of processing resources through sustained attention^37,78^. Thereby, LPC amplitude differences do not display a consistent direction in comparisons between emotional and neutral words. Most of the time, allocation of processing resources is higher for emotional than neutral stimuli^35,36^. However, this effect may reverse^87^, as allocation is strongly adaptive to task requirements^88^. In the current data, larger LPC amplitudes for emotional words presumably originate from intensified late processing caused by their higher salience. Larger LPC amplitudes of abstract compared to concrete words, however, could be linked to amplified late semantic processing caused by slowed lexico-semantic retrieval at earlier stages. Thus, the significant LPC interaction possibly reflects sustained efforts in the processing of abstract and emotional words at late processing stages. This may result in particularly low availability of late processing resources for neutral concrete words, as reflected in a comparatively small LPC amplitude. An initially slowed retrieval of abstract semantics may be compensated for by a subsequently increased allocation of processing resources. This effect might be especially pronounced in emotional abstract words. A resource-oriented interpretation of the LPC could also incorporate the increased allocation of resources to late elaboration in mental imagery. Notably, the interaction in the current study was most pronounced at left anterior electrode sites. This would be in line with the hypothesized origin of the LPC in the IFG.

In the fMRI data, no interaction occurred in a region typically involved in mental imagery or emotion processing. Rather, interactions were observed in the pars triangularis of the left IFG and in the left MTG. The left IFG and MTG are integral parts of the functional neuro-anatomical network for the semantic processing of words^17,89^. The MTG is involved in storage and activation of lexical information as well as their integration with semantic context. The IFG provides strategic, top-down control of lexical-semantic retrieval (anterior IFG) and selection between competing semantic alternatives (posterior IFG)^17^. Therefore, our findings presumably reflect varying activation within the semantic processing system. Similar, cross-over interactions were observed in both clusters: the emotion effect of the BOLD signal was inversed when comparing concrete and abstract words. Surprisingly, this suggests that emotional valence facilitates semantic retrieval of abstract words, but elicits a higher need for top-down semantic control in concrete words.

We therefore propose that emotional valence may, under specific circumstances, lead to additional processing load in the semantic selection process. This may happen if word properties other than emotional valence provide sufficient cues for the retrieval of lexico-semantic information. In concrete words, a sufficiently high number of lexico-semantic retrieval cues is available at early processing stages. Thus, subsequent semantic retrieval triggered by emotional cues may lead to a surplus of semantic information. Top-down controlled selection between semantic competitors may then be necessary at later processing stages. Regarding MTG and IFG activation, this would be reflected by the significantly more positive BOLD signal change following emotional concrete words than neutral concrete words. In the case of abstract words, comparatively few retrieval cues are available at early stages of word processing. Therefore, retrieval cues such as emotional connotation may facilitate semantic retrieval at later stages. This would reduce the need for top-down semantic control in emotional abstract compared to neutral abstract words. Here, the effect would be reflected by the significantly less positive BOLD signal change for emotional abstract than neutral abstract words. Notably, the temporal delay in the processing of abstract words (also visible through the delayed occurrence of the early posterior negativity (EPN)^28^) seems to play a key role in understanding this type of inverse effect of emotion. Nakic *et al*.^42^ reported that the IFG reacts quite sensitively to variable “selection load”, with negative valence influencing IFG activation dependent upon word frequency. This reinforces the notion that the amount of semantic information available early on may influence the integration of valence in semantic processing. Similar cross-over interactions between emotion and concreteness have been reported in performance of a semantic categorization task^90^ and a concreteness judgement task^54^. Interestingly, there also is evidence for a non-linear relationship between semantic context activation and the N400 amplitude. Abstract words elicit similar N400 amplitudes as concrete words that have a high number of semantic associates^20,21^.

Regarding our a priori hypotheses, the current data offer evidence in favor of grounded cognition. fMRI as well as ERP data support facilitated retrieval of emotional abstract words, especially at late, top-down controlled processing stages. This is consistent with the behavioral finding that neutral abstract words are especially prone to erroneous processing. Emotional valence may compensate for initially slowed processing of emotional abstract, but not neutral abstract words. Nevertheless, this interpretation is in need of further corroboration regarding the LPC (specifically, whether the modulation of this component indeed reflects allocation of processing resources) and the seemingly detrimental effect of emotional grounding on concrete words. It seems unlikely that the observed MRI interaction is based on differential degrees of mental imageability^9^. Imageability widely differs between emotional concrete and neutral abstract words, which contradicts similar BOLD signal changes for the two types of words. Moreover, the IFG does not seem to be involved in modality-specific visual processing^91^. Nevertheless, it is possible that visual semantic information is involved in emotion-dependent semantic processing. Abstract words can be represented through the building of complex visual scenes^92^. It is possible that such processes facilitate the processing of emotional abstract words, e.g. through enhanced retrieval of visual semantics. Independent of focusing on verbal or sensory aspects of semantic information, a profound understanding of the observed interaction pattern requires an understanding of the apparently heightened need for semantic control following emotional concrete words. It is important to clarify whether emotional cues influence the selection between semantic competitors or evoke additional retrieval of semantic information. Given the current results, investigating the threshold of lexico-semantic information that is necessary for sufficient semantic processing provides the most sensible starting point for this undertaking.

### Conclusion

We have demonstrated that emotional valence significantly modulates semantic processing as a function of word concreteness. The ERP data aligne this effect to late stages of word processing, that is, stages of elaborated semantic processing. The fMRI data imply that emotion modulates semantic processing in a top-down, controlled, highly context-sensitive manner. With regard to grounded cognition theories, it would be of interest to know how this kind of cortical, higher-order modulation differs from stimulus-driven, subcortical influences (for example, amygdala activation in response to written emotional words^44^). With respect to concreteness theories, our findings emphasize the importance of semantic control mechanisms. Current explanations of the concreteness effect^8^ do not include assumptions about controlled adaptation to the wider semantic context, for example, emotional connotation. Behavioral abstractness effects^25^ underline the relevance of mechanisms that potentially compensate for otherwise slowed processing of abstract words. In summary, it seems more important to ask how, and not whether, internal sensorimotor associations and affective states provide experiential grounding for a semantic concept. Our data show that the semantic system is sensitive to emotional retrieval cues as well as to word concreteness, and further, that it engages in complex higher-order semantic control when integrating both. The ways in which different kinds of sensorimotor and emotion grounding are mediated, integrated, and controlled remain subject to discussion.

## Supplementary information

Supplementary information.
