# Supplementary material for: Differential Impact of Emotion on Semantic Processing of Abstract and Concrete Words: ERP and fMRI Evidence
Source: Sci Rep. 2019 Oct 8;9:14439. doi: 10.1038/s41598-019-50755-3 (PMC6783415; doi:10.1038/s41598-019-50755-3)
Supplement: Supplementary file 1 — Supplementary information. [file 41598_2019_50755_MOESM1_ESM.docx]

Pauligk, Kotz & Kanske - **Differential Impact of Emotion on Semantic Processing of Abstract and Concrete Words: ERP and fMRI Evidence**

**Supplementary Information**

**1. Rating and Stimuli**

In the following section, the preparation of the stimulus material is described in more detail. The word material is identical to that used by Kanske & Kotz^1^.

In order to obtain concrete and abstract as well as negative, neutral and positive words, a rating experiment was conducted prior to the main study in which 720 German nouns were evaluated^2,3^. 40 students (20 female) from the University of Leipzig volunteered in the rating experiment and were paid for their participation. All participants were native speakers of German. The rating consisted of three parts, (1) a concreteness and (2) a valence rating. To also check for additional influences of arousal, an (3) arousal rating was conducted after the main experiment (30 participants, 15 female). The order in which the concreteness and the valence rating were presented was counterbalanced across participants. In both ratings, participants evaluated the words on a 5-point scale (concrete–rather concrete–neither/nor–rather abstract–abstract or negative– rather negative–neutral–rather positive–positive). Key function mapping was counterbalanced across participants. Stimuli were presented in the center of a computer screen. Participants had to respond within 2000 ms. 240 most consistently rated words were chosen for the main experiment. An equal proportion of each grammatical gender (female, male, neuter) was present in the sample. Word length varied between 4 and 8 letters. Frequency of usage was taken from the Wortschatz Lexikon of the University of Leipzig (<http://wortschatz.uni-leipzig.de/>). Familiarity of all stimuli was derived from the dlex database, a cooperative project of the University Potsdam and the project Digital Dictionary of the German at the Berlin-Brandenburg Academy of Science (http://www.dlexdb.de/). Half of the words were rated as very abstract, the other half as very concrete. One fourth was rated as very negative, another fourth as very positive and one half as neutral. The means and standard deviations (SD) for the words are listed in Supplementary Table 1. The words were selected such that an ANOVA yielded significant effects for valence (F(7,232)=1718.7, P<0.05), arousal (F(7,232)=149.2, P<0.05) and concreteness (F(7,232)=718.6, P<0.05), but not for frequency of usage or word length. Post- hoc comparisons (Scheffé test) yielded significant differences in valence between negative, positive, and the neutral word groups, in arousal between the two emotional groups vs. the neutral word groups, and in concreteness between all concrete vs. all abstract words (P<0.05 for all). All other comparisons were not significant (P>0.05). The four relevant groups of words (concrete emotional, concrete neutral, abstract emotional, abstract neutral) did not differ with regard to absolute (F(3, 236) = .44, P = .72 ) or rank familiarity (F(3, 236) = .50, P = .69). 240 pseudowords were created from the 240 words by changing one letter in each word following the phonological rules of German. Vowels were only replaced by vowels and consonants by consonants. The changed letter was always the first of a syllable. The position of the changed syllable (first, second or third) was balanced according to the different word lengths. The same set of words was used for the EEG and fMRI experiment.

|  | Word group | Example word / pseudoword | Rated valence | Rated concreteness | Rated arousal | Frequency | Word length | Familiarity |
| --- | --- | --- | --- | --- | --- | --- | --- | --- |
| Concrete | Negative | Feind / Beind *enemy* | 1.32 (0.52) | 4.40 (0.39) | 3.37 (0.12) | 12.30 (1.93) | 6.20 (1.19) | 112.25 (38.82) |
|  | Positive | Blume / Bluke *flower* | 4.52 (0.52) | 4.49 (0.49) | 3.19 (0.21) | 11.63 (1.99) | 5.60 (1.40) | 80.61 (28.56) |
|  | Neutral (1) | Kabine / Kazine *cabin* | 3.05 (0.36) | 4.69 (0.26) | 1.49 (0.07) | 12.26 (1.87) | 6.00 (1.05) | 144.14 (45.92) |
|  | Neutral (2) | Blech / Klech *plate* | 3.07 (0.26) | 4.71 (0.22) | 1.53 (0.13) | 12.00 (1.93) | 5.90 (1.18) | 39.20 (8.61) |
| Abstract | Negative | Gewalt / Gedalt *violence* | 1.18 (0.38) | 1.38 (0.21) | 3.61 (0.09) | 11.67 (1.97) | 5.80 (1.13) | 61.82 (16.88) |
|  | Positive | Wonne / Ponne *delight* | 4.68 (0.58) | 1.43 (0.26) | 3.21 (0.15) | 11.60 (1.77) | 6.16 (1.29) | 71.39 (31.78) |
|  | Neutral (1) | Optik / Opsik *optics* | 3.10 (0.50) | 1.68 (0.38) | 1.63 (0.27) | 12.16 (1.72) | 6.40 (1.10) | 67.03 (17.27) |
|  | Neutral (2) | Brauch / Krauch *costum* | 3.09 (0.25) | 1.71 (0.35) | 1.48 (0.20) | 11.66 (2.04) | 6.23 (1.17) | 84.62 (27.57) |
| Supplementary Table 1: **Properties of the word material**. Mean ratings and standard deviations (in parenthesis) for valence, arousal, concreteness as well as mean word frequency,number of letters and familiarity for the different word groups. The two neutral word groups correspond to those neutral words presented in a block with negative (1) and those presented in a block with positive (2) words. Example words are given together with the corresponding pseudoword and an English translation (in italics). | | | | | | | | |

**2. EEG effects of region and hemisphere**

**P2**

There was no significant effect of hemisphere (F(1,26)=.92, P=.35, η2=.03) or region (F(1,26)=1.49, P=.23, η2=0.06). There was a significant interaction between hemisphere and region (F(1,26)=6.04, P<.05, η2=.19).

**N400**

There was no significant effect of hemisphere (F(1,26)=3.61, P=.07, η2=.12). A significant effect of region was found (F(1,26)=14.18, P<.001, η2=0.35). The N400 amplitude was larger over anterior compared to posterior sites. There was no significant interaction between hemisphere and region (F(1,26)=.62, P=.44, η2=.02).

**LPC**

There was no significant effect of hemisphere (F(1,26)=.06, P=.82, η2=.00). There was a significant effect of region (F(1,26)=14.54, P<.01, η2=0.36) as LPC amplitude was larger over posterior than anterior electrode sites. There was no significant interaction between region and hemisphere (F(1,26)=.23, P=.64, η2=.01).

**3. EEG analysis including the central electrodes**

When analyzing the data we noted a central peak of the concreteness effects and therefore ran modified ERP analyses which included the central electrodes (posterior region: OZ, POZ, PZ, CPZ; anterior region: FCZ, FZ, AFZ, FPZ) and, thus, did not enclose the factor hemisphere. This analysis included the factors emotion (emotional, neutral), concreteness (concrete, abstract) and region (anterior, posterior). Because of the identical patterns of results (see below), we have chosen to report the set-up with larger spatial coverage and better comparability to our previous work in the main text.

**P2**

Analysis of the mean amplitude yielded a main effect of emotion (F(1, 26)=6.59, P<.05, η^2^=0.20) with larger amplitudes for emotional than neutral words.

**N400**

Analyses yielded a main effect of concreteness (F(1, 26)=28.50, P<.001, η^2^=0.52), as concrete words led to a larger N400 than abstract words. There was a significant interaction of emotion and region (F(1, 26)=18.64, P<.001, η^2^=0.42). The effect of emotion was significant at anterior electrode-sites (F(1, 26)=13.68, P=.001, η^2^=0.35), with larger N400 amplitudes elicited by neutral than by emotional words. There was no significant effect of emotion at posterior electrode sites (F(1, 26)=1.10, P=.30, η^2^=0.04).

**LPC**

The LPC was larger for abstract than for concrete words (F(1,26)=22.57, P<.001, η^2^=0.47). Emotional words yielded a higher LPC amplitude than neutral words (F(1,26)=23.93, P<.001, η^2^=0.48). There was a strong trend towards an interaction between emotion and concreteness (F(1,26)=3.77, P=.06, η^2^=0.13), indicating that the effect of emotion may be larger for concrete than for abstract words. Also, emotion interacted with region (F(1,26)=6.63, P<.01, η^2^=0.20).

Following up on the significant influence of region as main effect and in interaction with emotion, the ANOVA was repeated for anterior and posterior electrodes separately. Results showed unaltered main effects and interactions in both regions, and, additionally, a significant interaction between emotion and concreteness in the anterior region (F(1,26)=9.49, P<.01,η^2^=0.27). At anterior electrode sites, the effect of emotion was larger for concrete (F(1,26)=32.19, P<.001, η^2^=0.55) than for abstract words (F(1,26)=5.30, P<.05, η^2^=0.17), yielding especially low LPC amplitudes for neutral concrete words.

**4. fMRI effects of Emotion and Concreteness**


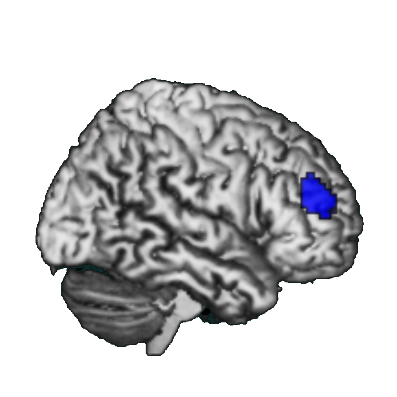

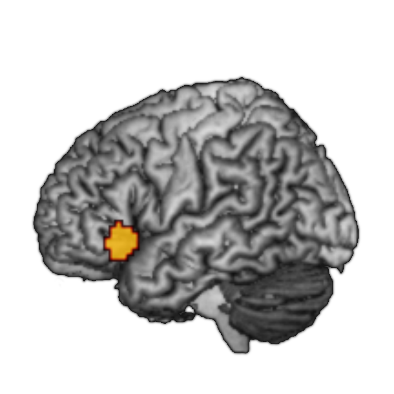


Supplementary Figure 1. **fMRI effects of emotion and concreteness.** Regions surpassing the maximum activation proportion expected under the null hypothesis superimposed on a standard single subject brain. Blue: Contrast Neutral > Emotional; Yellow: Contrast Abstract > Concrete.

**References**

1. Kanske, P. & Kotz, S. A. Concreteness in emotional words: ERP evidence from a hemifield study. *Brain Res.* **1148,** 138–148 (2007).

2. Kanske, P. & Kotz, S. A. Leipzig Affective Norms for German: A reliability study. *Behav. Res. Methods* **42,** 987–991 (2010).

3. Kanske, P. & Kotz, S. A. Cross-modal validation of the Leipzig Affective Norms for German (LANG). *Behav. Res. Methods* **43,** 409–413 (2011).
